# Dr. Carswell

**Published:** 1841-01

**Authors:** 


### DR. CARSWELL.

THIS distinguished pathologist has recently been appointed to the honorable and important office of physician to the King of the Belgians. We have much pleasure in giving a place in our pages to the following highly complimentary address presented to him on resigning his chair in University College. We entirely concur in the sentiments contained in it, as we believe that the profession does not contain a more honest or more successful cultivator of pathological science, or a more honorable man, than Dr. Carswell.

*To* ROBERT CARSWELL, M.D., *Professor of Pathological Anatomy in University College, London, and Physician to University College Hospital, &c. &c.*

Sir,—The students of the faculty of medicine of University College have learnt with much regret that they are about to lose the advantages which they have long derived from your services as professor of pathological anatomy, and as one of their clinical instructors. The painful feeling with which they contemplate your separation from them is, however, accompanied with all the satisfaction it admits of, by their being enabled to indulge the hope and the belief that the change you are about to make is calculated to prove advantageous to yourself, more especially in the prospect it affords to you of a degree of health which they have observed with sorrow to be apparently inconsistent with the discharge of the laborious and responsible duties with which you have been intrusted.

They cannot now permit you to depart without offering you their most earnest assurance how highly they appreciate the honour of having had for their teacher one who has contributed so much to the advancement of scientific medicine. They look back with gratitude and pleasure to the valuable information they have derived from your labours, and to the rare and high example of a disinterested love for science and devotion to its interests, which your conduct has afforded them. Of your private virtues they forbear to say much; your honorable sentiments and conduct, your unsullied integrity, the urbanity of your manner, and your eminently disinterested kindness are familiar to all who have had the least acquaintance with you; and they desire to assure you, that, however unostentatious and unobtrusive have been the acts by which these feelings have been manifested, they have nevertheless not passed unobserved, and will not be unremembered.

They beg to offer you their warm congratulations on the evidence afforded by your present appointment, that your merits have been appreciated by a sovereign as much distinguished by his moral and intellectual endowments as by his illustrious alliances and high station; and they now take leave of you, with the expression of their sincere desire that the change you are about to make may be productive of all the advantages you expect from it, and, especially, that it may lead to the perfect restoration of your health, and the enjoyments inseparable from health, which they know you have sacrificed in their service. Wherever you are, be assured you will be attended by their grateful remembrances, and by their deeply-felt wishes for your future prosperity and happiness.

October 20, 1840. JOHN TAYLOR, *Chairman.*

(REPLY.)

*To the Students of the Medical Faculty of University College.*

Gentlemen,—It gives me the most unfeigned pleasure to receive from you such a public testimony of your kindness and esteem; and to have it thus made known to me, that I part from you with the assurance that I carry with me your sincere wishes for my health, my future prosperity, and happiness.

The honour, gentlemen, has been mine—that of having been your teacher; and, if you look back with gratitude and pleasure to the valuable information you say you have received from my labours, and which, in your opinion, have contributed so much to the advancement of scientific medicine, the more do I feel myself bound to express my gratitude to you, and to avow that your approbation of my humble merits is, indeed, no small reward for whatever sacrifice I may have made to the attainment of such gratifying and important results.

Be assured, gentlemen, that your opinion of my character in my social relations and intercourse with you, for a number of years, is the more pleasing to me that it marks a prominent feature in your own character, by which you are distinguished among all others of the same class as yourselves, and to which I feel justified in attributing much of the success and distinctions you have acquired by your professional industry and talent.

Accept, gentlemen, the expression of the same feelings and sentiments for your prosperity and happiness, which you have so warmly manifested for mine; and be assured that I shall always feel as deep an interest in your welfare as in the success and advancement of that institution under whose liberal and enlightened influence you have commenced your useful and honorable career, and in which I have had the proud honour of being enrolled among its professors.

ROBERT CARSWELL.

